# The risk factors of low anterior resection syndrome after colorectal cancer surgery: A retrospective study of 566 patients in a single institution in China

**DOI:** 10.3389/fsurg.2022.990702

**Published:** 2022-08-25

**Authors:** HyokJu Ri, HaoNan Kang, ZhaoHui Xu, KunHyok Kim, YanYing Ren, ZeZhong Gong, Xin Chen

**Affiliations:** ^1^ Department of Hernia and Colorectal Surgery, The Second Affiliation Hospital of Dalian Medical University, Dalian, China; ^2^ Department of Colorectal Surgery, The Hospital of Pyongyang Medical College, Pyongyang, Democratic people's republic of Korea, Korea; ^3^ Department of Pathophysiology, The University of Hamhung Medical College, Hamhung, Democratic people's republic of Korea, Korea

**Keywords:** low anterior resection syndrome, colorectal cancer, total mesorectal excision, sphincter-preserving, risk factor

## Abstract

**Purpose:**

This study aims to identify the independent risk factors in the low anterior resection syndrome (LARS) after surgery for colorectal cancer (CRC).

**Method:**

This was a retrospective, single-institution study in the Second Affiliation Hospital of Dalian Medical University, China. Patients underwent sphincter-preserving low anterior resection with total or partial mesorectal resection (with or without protective ileostomy) and completed a self-filled questionnaire over the phone to assess postoperative bowel dysfunction from January 2017 to December 2019. The predictors of LAR were evaluated using univariate and multivariate analyses.

**Result:**

The study population was 566 patients, 264 (46.64%), 224 (39.58%), and 78 (13.78%) patients with no, minor, and major LARS, respectively. In the univariate analysis, independent factors such as tumor location and size, anastomotic height, protective ileostomy, post-operation chemoradiotherapy, tumor T stage, lymphatic nodal metastasis classification, surgery duration, and time interval for closure of stoma were significantly associated with LARS points while we found the tumor T stage and lymphatic nodal metastasis classification as the new independent risk factors compared with the last decade studies. In the multivariate analysis, factors such as low and middle tumor location and protective ileostomy, and post operation treatment, nodal metastasis classification were the independent risk factors for major LARS.

**Conclusion:**

The new independence risk factors were tumor T stage and lymphatic nodal metastasis status in univariate analysis in our study, with anastomotic height, low and middle tumor location, protective ileostomy, post-operation chemoradiotherapy, nodal metastasis status increasing LARS point in multivariate analysis after surgery for CRC.

## Highlights

Low anterior resection syndrome (LARS) is a common functional bowel disorder that develops after anal-sparing rectal cancer surgery.

The LARS score was developed to allow an assessment of the syndrome.

A few studies have attempted to identify LARS risk factors, but have generally failed to comprehensively report the statistical significance of the factors identified.

This is important to raise awareness among clinicians and researchers to focus on this syndrome, to improve prevention and treatment of bowel disorders such as LARS, as well as to inform patients.

## Introduction

CRC is the third most common cancer in the world, accounting for more than one third of all cancers, with an age-standardized rate of 7.7 per 100,000, and among them the rectal cancer is accounting for 5% in all cases (1–4). With the advances in chemotherapy, radiotherapy, and surgical techniques, the long-term survival rate is increasing after CRC surgery regardless of the rising incidences of these diseases (5–7).

The major surgical procedure for rectal cancer involves abdominoperineal resection (APR or called as Mile's procedure) and low anterior resection (LAR) with preservation of sphincter muscles. In recent years, LAR with total mesorectal excision (TME) is the gold standard in rectal cancer surgery (8, 9). LAR and partial mesorectal resections are the most appropriate surgical procedures for upper rectal cancer (1, 5, 10, 11). Laparoscopic LAR is a technically difficult procedure that involves transection of the intraperitoneal rectum in a limited pelvic cavity, and the undesirable result of this surgery is low anterior resection syndrome (LARS). The prevalence is around 80%–90%, and patients experience LARS with varying degrees of severity after sphincter-preserving LAR surgery (1–3, 9, 12, 13).

The conception of LARS is hard to define and involves some altered evacuation status after LAR. It can be described as a “disordered bowel function after rectal resection, leading to a detriment in quality of life.” (14–17). The etiology of LARS is poorly understood, and it seems that the anatomical components and physiological functions of normal defecation, which may be damaged during surgery, are not well established (9, 18). The colorectal experts established LARS scoring system which had five-item validated questionnaire evaluating the bowel functions after CRC surgery in 2012, and this questionnaire has been used to evaluate LARS worldwide. (Table 1) (5, 19, 20). They also focused to find the risk factors influencing LARS happening, and many studies reported the several risk factors for predicting the severity of LARS. Unfortunately, they had some limitations of sample size and insufficient following up. Therefore, we think it is important to identify the risk factors of LARS using comprehensive understandable scoring system and prevent this undesirable result of CRC surgery.

**Table 1:** LARS scoring system questionnaire.

1. Do you ever have occasion when you cannot control your flatus (wind)?	
No, never	0
Yes, less than once per week	4
Yes, at least once per week	7
2. Do you ever have any accident leakage of liquid stool?	
No, never	0
Yes, less than once per week	3
Yes, at least once per week	3
3. How often do you open your bowels?	
More than 7 times per day (24 h)	4
4–7 times a day (24 h)	2
1–3 times a day (24 h)	0
Less than once a day	5
4. Do you ever have to open your bowels again within 1 h of the last bowel opening?	
No, never	0
Yes, less than once per week	9
Yes, at least once per week	11
5. Do you ever have such a strong urge to open your bowels that you have to rush to the toilet?	
No, never	0
Yes, less than once per week	11
Yes, at least once per week	16
	0–20: No LARS 21–29: Minor LARS 30–42: Major LARS

In this study, we tried to identify the independent risk factors influencing LARS after rectal cancer resection based on the recent database for the advanced research.

## Materials and method

### Type of study

This was a retrospective study with prospectively collected information from the Second Affiliation Hospital of Dalian Medical University in China. All the patients were diagnosed with CRC and underwent sphincter-preserving LAR with intensive treatments from January 2017 to December 2019.

### Population of study

#### Inclusion criteria

Any patient diagnosed with CRC and underwent LAR was included in this study without any age or gender specifications, and tumor location ranging from 5–25 cm off the anal verge. All patients underwent colonoscopy, CT (or MRI test if necessary) and other tests, and were diagnosed as rectal cancer.

#### Exclusion criteria

1. Patients with unresectable cancers.
2. Patients assessed as more than ASA grade 3.
3. Patients with poor-quality total mesorectal excision (TME) surgery or breached circumferential tumor margins in complete mesocolic excision (CME) surgery.
4. Patients who underwent abdominal perineal resection (APR, also called as Miles procedure) or proctosigmoidectomy (Hartmann procedure).
5. Patients who did not complete the LARS questionnaire or follow-up.
6. There was no pediatric patient in our study.

#### Endpoints (outcome parameters)

Every patient was followed up for more than one year after LAR surgery and filled a LARS score questionnaire. The endpoint was the completion of the analysis in January 2021.

#### Operation

All resections were performed by five of professionally-certified and fellowship-trained colorectal surgeons, who all shared a similar case volume over the study years.

### LARS questionnaire and data collection

LARS questionnaire was used for assessing the bowel function and included the following items: flatus incontinence, liquid stools status, frequency, clustering, and urgency. Every item has three options with a defined scoring system used for evaluating the severity. The patients were divided into the no (0–20), minor (21–29), and major (30–42) LARS groups depending on their total score (Table 2).

**Table 2:** Distribution of patients according to LARS score and study variables (*n* = 566).

Variable	Level	LARS			*p* value
		No LARS	Minor LARS	Major LARS	
Gender	Male	178	136	40	0.066
	Female	86	88	38	
Age		64.50 ± 13	64.00 ± 14	64.00 ± 17	0.724
BMI		23.68 ± 2.89	23.82 ± 3.09	23.58 ± 3.63	0.776
Tumor location	Low	10	48	54	<0.001*
	Middle	118	164	24	
	High	100	8	0	
	Sigmoid	36	4	0	
Tumor size		3.00 ± 3.00	4.00 ± 2.00	4.00 ± 2.1	0.028*
Anastomotic height		11.98 ± 4.40	7.31 ± 2.39	5.26 ± 1.27	<0.001*
Operation type	Laparotomy	10	16	26	0.102
	Laparoscopy	254	208	52	
Protective ilesostomy	Yes	174	54	6	<0.001*
	No	90	170	72	
Pre-operation treatment	Yes	40	80	40	0.081
	No	224	144	38	
Post-operation treatment	Yes	168	116	36	0.020*
	No	96	108	42	
T stage	T1	16	14	2	0.009*
	T2	26	36	28	
	T3	100	80	22	
	T4	122	94	26	
Nodal classification	N0	170	136	48	0.059
	N1	74	66	22	
	N2	20	22	8	
Metastasis	M0	240	208	76	0.211
	M1	24	16	2	
Operation time		175.00 ± 65.99	187.50 ± 78.45	180.00 ± 93.83	0.005*
Time interval to close ileostomy		41.61 ± 75.96	102.16 ± 89.82	135.56 ± 106.71	<0.001*

* Significant differences between the LARS subgroups*.*

We used the Chinese version of the questionnaire. Patient demographics, pre-and-post operative data, surgery information, and pathological data were obtained from the hospital database, and the three groups were compared. We measured the tumor location using the specimen from the anal verge after surgery, and the tumor location was divided into four degrees, such as low (=<5 cm), middle (5–10 cm), high (10–15 cm), and sigmoid (>15 cm). The anastomotic height was measured based on the tumor location and operation procedure in the surgery. The cancer stage was defined using the 8th edition American Joint Commission on Cancer (AJCC) Tumor Node Metastasis (TMN) classification system. In this study, the pathological stage was defined as the cancer stage after surgery.

### Follow up

LARS scores were assessed for more than one year after an operation during follow-up. In this study, patients received phone calls and explained the questionnaire in detail, and they were asked to complete a validated Chinese version of the questionnaire designed to evaluate LARS score after CRC surgery. We rechecked the addresses and phone numbers for the patients who did not receive the calls, then reminded them or their family members to complete the questionnaire. The follow-up process was completed over three months.

### Last decade studies database

We searched the PubMed (“Title/Abstract” add to the query box) and Web of Science Core Collection database (“TI = Title” and “AB = Abstract” add to the query box) from January 2011 to December 2021, using a combination of relevant Medical Subject Heading terms and keywords: (low anterior resection* OR LAR* OR low anterior resection syndrome* OR LARS*) AND (risk factor* OR independent factor* OR independent risk factor* OR quality of life* OR QoL*) AND (rectal cancer* OR colorectal cancer* OR colon cancer*) AND (surgery* OR operation* OR resection*). And we selected the most citied and suitable 21 papers, which researched about the risk factors of LARS among the 3,450 papers (642 papers from PubMed, 2,808 papers from Web of Science), and summarized their risk factors reported before.

### IRB approval/ethics

The Ethics Committee of the Second Affiliation Hospital of Dalian Medical University approved this study. All patients were given information regarding the surgery and informed consent was obtained before surgery.

### Statistical analysis

All data collection and statistical analyses were performed using EndNote 20.0, Excel 2019, and Social Science SPSS Advanced Statistics 26.0 (IBM Software Group). The mean, standard deviation, and median values (interquartile range) were used to describe the normal and non-normal distribution measurement data. Frequency (percentage) was used to describe the classification data. The one-way ANOVA and nonparametric tests were used to compare the measurement and classification data between the groups. Statistical significance was set at *p *< 0.05.

First, we used univariate analysis to find factors with significant associations with LARS. Then, we performed the multivariate analysis with the variables representing significant differences in the univariate analysis. We confirmed the risk factors associated with LARS using the ordered logistic regression analysis.

## Result

**Figure 1:**
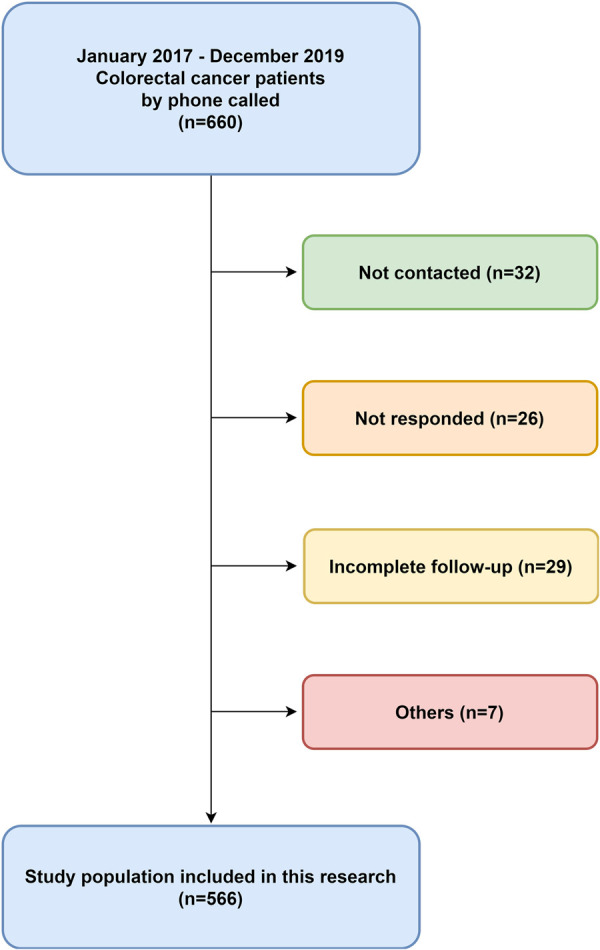
Patients collection diagram. This was a retrospective, single-institution study with colorectal cancer who had undergone low anterior resection from January 2017–December 2019.

We collected 660 patient data from the hospital database, and 566 patients responded completely (85.76%). Among the 660 patients, 32 could not be contacted, 26 did not respond, 29 returned incomplete questionnaires, and seven died because of several causes, including the other diseases or accidents. We excluded these 94 patients from the analyses. Therefore, the study population was 566 patients with 354 men and 221 women (Figure 1).

The mean age was 63.44 y (64.60 y and 61.50 y for men and women, respectively). The median follow-up was 15.6 months (10–22 months) after surgery. Depending on the LARS score, we divided the patients into the no LARS, minor LARS, and major LARS groups with 264 (46.64%), 224 (39.58%), and 78 (13.78%) patients, respectively. Laparoscopic surgery and protective ileostomy were performed on 514 (90.81%) and 354 (41.34%) patients, respectively. There were 10 incidences of anastomosis leakage (1.8%) (Table 2).

The results of the univariate analysis are shown in Table 3. LARS was significantly more frequent in patients with factors, such as low tumor location and tumor size, protective ileostomy versus no ileostomy, post-operation chemoradiotherapy, tumor T stage, nodal, long surgery duration, and time interval between ileostomy closure. In contrast, gender, age, BMI, surgery type (laparoscopy or open procedure), pre-operation chemoradiotherapy, and tumor metastases were not associated with LARS development.

**Table 3:** Result of univariate analysis.

Variable	Level	Odds ratio (95% confidence interval)	*p* value
Gender	Male	1.163 (0.066–1.333)	0.519
	Female	1	
Age		0.97 (0.94–1.0)	0.628
BMI		1.589 (1.272–2.278)	0.120
Tumor location	Low	1.293 (1.009–1.577)	<0.0001*
	Middle	0.593 (0.334–0.852)	
	High	1.026 (1.011–1.259)	
	Sigmoid	1	
Tumor size		0.069 (0.032–0.363)	<0.0001*
Anastomotic height		0.67 (0.59–0.75)	<0.0001*
Operation Type	Laparotomy	1.333 (1.052–2.719)	0.471
	Laparoscopy	1	
Protective ilesostomy	Yes	1.664 (1.513–1.863)	<0.0001*
	No	1	
Pre-operation Treatment	Yes	1.338 (1.836–2.519)	0.182
	No	1	
Post-operation Treatment	Yes	0.139 (0.028–0.358)	0.022*
	No	1	
T stage	T1	1.041 (0.322–1.403)	0.001*
	T2	0.419 (0.181–0.657)	
	T3	0.911 (0.194–1.173)	
	T4	1	
Nodal classification	N0	1.105 (0.193–1.402)	0.014*
	N1	1.081 (0.238–1.400)	
	N2	1	
Metastasis	M0	0.81 (0.525–1.104)	0.188
	M1	1	
Operation time		1.476 (1.336–2.336)	0.038*
Time interval to close ileostomy		3.131 (0.742–3.258)	0.002*

* Significant differences*.*

In the multivariate analysis, the independent risk factors related with LARS were anastomotic height, low and middle tumor locations, nodal classification and protective ileostomy (Table 4).

**Table 4:** Result of multivariate analysis.

Multivariate and levels		Odd ratio (95% *CI*)	*p* value
Tumor size (diameter)		1.02 (0.86–1.20)	0.813
Operation time		1.00 (0.997–1.004)	0.705
Time intervals to close stoma		1.003 (0.999–1.006)	0.132
Anastomotic height		8.028 (4.428–21.714)	<0.001*
Tumor location	Low	80.39 (15.21–424.54)	<0.001*
	Middle	11.03 (2.33–56.25)	0.002*
	High	0.86 (0.13–5.46)	0.871
	Sigmoid	1	
Protective Stoma (ileostomy)	Yes	0.35 (0.16–0.79)	0.01*
	No	1	
Postoperation Treatment	Yes	1.39 (0.79–2.40)	0.047*
	No	1	
T stage	T1	0.87 (0.25–3.02)	0.824
	T2	1.84 (0.85–3.98)	0.121
	T3	0.85 (0.46–1.56)	0.594
	T4	1	
Nodal classification	N0	0.715 (0.193–1.102)	0.022*
	N1	0.981 (0.238–1.336)	0.038*
	N2	1	

* Significant differences.

## Discussion

Sphincter-preserving low anterior resection (LAR) improves the quality of life (QoL) of patients with middle and low colorectal cancer, and several large randomized clinical trials have reported the safety and feasibility of this procedure (21, 22). Therefore, it has become a popular treatment method (23, 24). However, the undesirable result of this procedure is the bowel dysfunction called low anterior resection syndrome (25). About 80% of patients who undergo this procedure experience varying degrees of LARS (26, 27).

LARS generally consists of fecal incontinence, urgency, and incomplete evacuation or evacuation difficulties. Several articles reported the leakage of gas and stool, stool clustering, frequent bowel movements, evacuation, and urgency as the main complaints (1, 7, 14, 17, 28, 29). LARS can have two types of symptoms, the first type appears within 6–12 months after surgery, which is called short-term symptoms. They are usually caused by short-lived neorectal irritabilities during the postoperative period, and includes fecal urgency, incontinence, and increased frequency. The second type extends for more than one year after surgery. They are called long-term symptoms and are most likely caused by constant changes, and includes constipation, feelings of incomplete excretion, and bowel-emptying difficulties (7, 10, 28). Some patients show characteristics of both types. They alternate between the two patterns or experience both at the same time (30–33). These symptoms are caused because of damage to several factors, such as nerves and muscles of defecation (18, 24, 34).

LAR surgery can injure components of the anal canal, such as the internal anal sphincter, longitudinal conjunctive muscle, or hiatus ligament, or can cause mechanical or nerve damage through injury to these organs. The resection of the rectum, division of the coccygeus muscle, and/or damage to the nerve supply can impair rectal function. The remaining rectum is small and does not function properly, and the hypermotility of the remnant colon can affect the manifestation of urge fecal incontinence (7, 16, 17).

The first idea for LARS scoring system came up in 1998, and the Memorial Sloan Kettering Cancer Center Bowel Function Instrument (MSKCC-BFI) created the 18 items validated scoring system in 2004 that can be used to assess the bowel function after LAR (35). This scoring system surveys several factors, including diet number, form, quality and timing of bowel movements, sensation of flatus, anti-diarrheal medication usage, and fecal incontinence. This scoring system ranged from 18–90, higher scores indicate better levels of bowel function. However, this scoring system was not universally applicable and could not be widely used (1, 14, 36). The second idea of LARS scoring system which had five-item validated questionnaire evaluating the bowel functions after CRC surgery in 2012, and this questionnaire has been used to evaluate LARS worldwide.

The risk factors of severe LARS are related to the anastomotic height, pre and postoperative chemoradiotherapy, anastomotic leakage, and protective ileostomy etc. (8, 9, 32, 37, 38).

In our study, we firstly identified the independent risk factors associated with LARS in univariate analysis, including tumor location and tumor size, anastomotic height, protective ileostomy versus no ileostomy, post-operation chemoradiotherapy, tumor T stage, nodal classification, long surgery duration, and time interval between ileostomy closure, while the tumor T stage and nodal classification were clarified as the new independent risk factors while the last decade studies have not reported.

When having low anterior resection procedure for CRCs, it takes time for the bowel to adapt after the operation, which helps in intestinal function recovery. And protective ileostomy was performed, the patients have difficulties controlling their defecation. The loss of bowel functions leads to stool defecation without consciousness, and this phenomenon adversely affects LARS recovery.

Tumor location, size, T stage and lymphatic nodal characteristics are directly related to surgical range and procedures; therefore, LARS is directly influenced by these three factors (39–41). But this theory is suggested in our study and the other studies have no mentioned the tumor T stage and nodal classification as the risk factors in their researches.

The side effect of neoadjuvant radiotherapy and chemotherapy is intestinal dysfunction, which is caused by nerve and muscle damage in the colon (38, 42–44). In 2017, L.M. Jimenez-Gomez et al., (8) reported risk factors, such as TME and neoadjuvant and adjuvant radiotherapy can increase the risk of major LARS. In 2020, Theresa H. Nguyen et al., (1) proved that neoadjuvant and adjuvant radiotherapy were risk factors for LARS, especially major LARS, even in patients with large rectal residuals. And several studies have shown that LARS is divided into incontinence-dominant and frequency-dominant modes. Each mode is associated with different risk factors. The incontinence-dominant mode is related to preoperative radiotherapy and postoperative complications. The frequency-dominant mode is related to the low tumor location from the anal margin; however, the overall main LARS is related to poor quality of life. The frequency-dominant type of LARS has a more profound impact on postoperative quality of life (10, 20, 45). In 2019, Keiji Koda et al., (18) showed that removing most of the rectum can damage the internal sphincter muscle and/or rectal wall, and deconstruct structures around the levator hiatus, are factors involved in the development of LARS symptoms.

In recent years, significant incidences of postoperative intestinal dysfunction and the prospects of a good prognosis have made radical resection plus neoadjuvant radiotherapy the standard treatment. However, there are some practical difficulties to perform the complete radical resection. In this theory, full-dose neoadjuvant chemotherapy can reduce tumor size similar to radiotherapy plus chemotherapy, reducing the possibilities of local recurrence in patients undergoing surgical resection. It also reduces the incidences of distant metastases. These studies have shown that neoadjuvant chemotherapy is usually an effective method for the treatment of locally advanced rectal cancer, and the effects are satisfactory (46, 47). Considering that neoadjuvant chemotherapy has no significant effect on bowel function, it may be a reasonable treatment option for major LARS patients (38).

In our study, only post-operative chemoradiotherapy was identified as a risk factor for severe LARS development in terms of neoadjuvant and adjuvant treatment of CRCs. We thought that this result came from the differences in treatments and conditions according to every country and national race.

In 2021, Suzuki, N et al., (48) also reported anastomotic complications, such as leakage, which was confirmed to be associated with a 3.5-fold increase in the incidences of major LARS. However, we could not find anastomotic complications increasing the incidences of major LARS in our study, and we thought this was due to the development of operation skills and reliable management of patients after operation in recent years.

Several studies have suggested an algorithm for the treatment of LARS, including conservative therapies, biofeedback, and sacral nerve stimulation. In 2019, Chirs George et al., (42) reported that conservative treatment (internal medicine, physical therapy, and trans-anal irrigation), invasive surgery (neuromodulation), and multimodal therapy were the main methods for treating LARS in patients. If these treatments were not working wonderfully, it's recommended to perform stoma surgery. The definitive stoma surgery was considered if major LARS persisted for more than 2 years (7, 24, 29). In 2021, K. Neumann et al., (49) found that transanal endoscopic microsurgery (TEM) for rectal tumors was associated with significantly reduced hospitalization costs, which far exceeded the cost of acquiring and maintaining the technology, and reduced the incidence of LARS, so recommended that if possible use TEM to treat rectal cancer.

**Figure 2:**
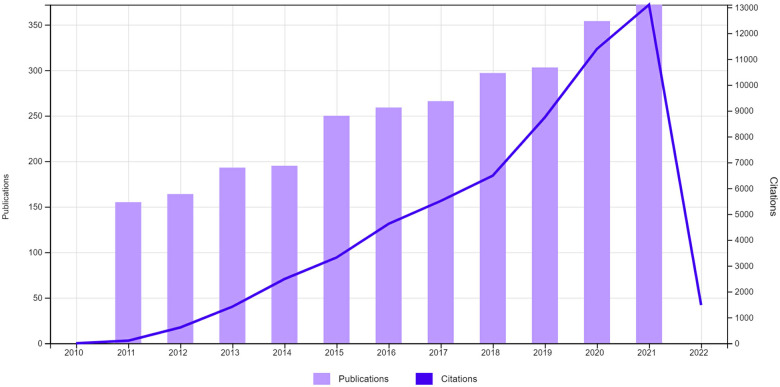
Paper publication and citation numbers in the last ten years (2011–2021).

When we are focusing on the number of articles published each year for the last ten years, the publications and citations trend to increase obviously (Figure 2: downloaded from Web of Science Core Collection). This shows that research for LARS and improving QoL is recently one of the major focuses in the colorectal fields as patient requests. And, the independent factors are similar to the others, including pre- and post-surgery chemoradiotherapy, poor TME procedure, tumor height from the anal verge, anastomosis height and leakage, temporary protective ileostomy, and complications after surgery (Table 5). We thought it would give a well-updated knowledge for future studies. We thought there are some limitations in our study such as not enough numbers of database, single institution study design and no mentions on LARS treatment. These can affect the undesirable effects on the study results and general ideas. We hope an updated and advanced study is needed for a better understanding to provide more information on LARS treatment strategies improving the quality of life.

**Table 5:** The independence risk factors of LARS.

Author	Country	Published Year	Type of the study	Sample size	Independence Risk Factors of LARS	Outcomes (OR, 95%CI value/*p* value/RR value)
Bregendahl, S. (50)	Denmark	2013	Retrospective, cross-sectional study	938	Neoadjuvant therapy	2.48 (1.73–3.55)
					TME procedure	2.31 (1.69–3.16)
					Age <64 years old	1.9 (1.43–2.51)
					Female gender	1.35 (1.02–1.79)
					Anastomotic leakage	2.06 (0.93–4.55)
Juul, T. (51)	Denmark, Spain, Sweden, Germany	2014	Retrospective multicentral study	801	Radiotherapy	<0.0001
					Mean tumor distance from anal verge	=0.003
Juul, T. (52)	Denmark + UK	2019	Retrospective, cross-sectional study	1875	Neoadjuvant chemoradiation	3.5 (1.15–9.4)
					Anastomosis height	=0.0001
Bondeven, P. (53)	Denmark	2015	Retrospective, cross-sectional study	125	Long course neoadjuvant chemotherapy	3.5 (1.15–9.4)
					Remnant rectum <4 cm	=0.0001
Wells, C.I. (54)	New Zealand	2015	Retrospective study	277	Low anastomosis height	2.11 (1.05–4.27); *p* = 0.04
					Obstructive presenting symptoms	6.71 (1.00–44.80); *p* = 0.05
					Post-operative chemotherapy (at 1 year)	1.93 (1.04–3.57); *p* = 0.03
					Temporary diverting ileostomy	2.49 (1.04–5.95); *p* = 0.04
Hain, E. (55)	France	2017	Retrospective study with prospectively collected data	135	Long course radiotherapy	=0.0007
					Anastomotic leakage	=0.02
					Hand-sewn anastomosis	=0.003
					Side-to-end anastomosis	=0.01
Carrillo, A. (56)	Spain	2016	Retrospective, cross-sectional study	195	Long course radiotherapy	=0.019
					TME (total mesorectal excision) / PME (partial mesorectal excision)	<0.001
					Protective ileostomy Yes / No	=0.003
					Coloplasty Yes (lower rate of LARS) / No (high rate of LARS)	0.017
Ekkarat, P. (37)	Thailand	2016	Retrospective study	129	Adjuvant radiotherapy (neoadjuvant excluded)	6.55 (2.37–18.15)
					Anastomosis height <5 cm	3.76 (1.34–10.61)
					Protective ileostomy	=0.024
Sturiale, A. (57)	Italy	2017	Retrospective study with prospectively collected data	93	Neoadjuvant radiotherapy	=0.04
					Tumor location from anal verge <5 cm	=0.003
					Age >70 years old	=0.003
					Time interval for closure of ileostomy	=0.002
Hughes, D.L. (58)	Wales	2017	Prospective clinical cohort study	65	Neoadjuvant radiation	<0.01
					Tumor location <8 cm	1.6 (0.6–4.1)
					Ileostomy close interval >1year	3.7 (1.1–13.1)
Battersby, N.J. (59)	Denmark	2018	Multicenter Cross-Sectional Study	578	Radiotherapy	<0.001
					Tumor height <5 cm	<0.001
Sarcher, T. (60)	France	2018	Review study	N/A	Neo-adjuvant treatment	RR = 2.48
					TME versus PME	RR = 2.31
					Anastomotic leak	RR = 2.06
					Female gender	RR = 1.35
					Age <64 years old	RR = 1.90
Nowakowski, M.M. (61)	Poland	2018	Prospective clinical cohort study	56	Preoperative radiotherapy	11.9 (2.98–47.48); *p* < 0.001
					Distance of the tumor from the anal verge	0.69 (0.55–0.86); *p* = 0.001
					Bowel preparation	6.27 (1.51–26.7); *p* = 0.01
					Protective ileostomy	15.97 (4.07–61.92); *p* = 0.001
Sun, W. (62)	China	2018	Single-center cohort of the randomized controlled trial	220	Long-course neoadjuvant radiation	2.20 (1.24–3.91); *p* = 0.007
					Height of anastomosis	0.74 (0.63–0.88); *p* = 0.001
					Diverting ileostomy	2.59 (1.27–5.30); *p* = 0.009
Nuytens, F. (63)	Belgium	2018	cross-sectional observational study	100	Postoperative radiotherapy	<0.04
Rubinkiewicz, M. (64)	Poland	2019	Prospective study	46	Post-operation comlication	=0.02
Miacci, F.L.C. (65)	UK	2020	Retrospective cohort study	64	Distance from the anastomosis to the anal margin	<0.001
					Neoadjuvant therapy	<0.0014
					Protective ileostomy	0.0023
Bolton, W.S. (66)	UK	2020	International, retrospective cohort study	132	Every 1 cm decrease in tumor height above the anal verge	1.290 (1.101,1.511)
					ASA grade >1	2.920 (1.239, 6.883)
Dulskas, A. (67)	Lithuania	2020	Single-center randomized controlled trial	43	Preoperative chemoradiotherapy	<0.001
Rizzo, G. (68)	Italy	2021	Retrospective study with prospectively collected data	113	Preoperative chemoradiotherapy	<0.012
Benli, S. (69)	Turkey	2021	Retrospective, clinical study	276	Very low anterior resection procedure	42.40 (11.14–161.36); *p* < 0.0001
					Protective ileostomy	12.83 (6.58–25.0); *p* < 0.0001
					End colostomy	8.55 (1.36–53.61); *p* = 0.022
					Chemotherapy	3.08 (1.71–5.53); *p* < 0.0001
					Radiotherapy	2.51 (1.38–4.57); *p* = 0.003
					Anastomosis location >8.5 cm	<0.05
Jiménez-Rodríguez, R. M. (70)	Spain	2021	Retrospective study with prospectively collected data	150	Male gender	*p* = 0.004 (1.00–4.64)
					Preoperative neoadjuvant therapy	*p* = 0.048 (1.48–7.74)

=/<***; *p* value, ** (**-**); OR (95% CI) value, **RR**; relative risk value, **N/A**; none.

## Conclusion

The new independence risk factors were tumor T stage and lymphatic nodal metastasis status in univariate analysis, while anastomotic height, low and middle tumor location, protective ileostomy, post-operation chemoradiotherapy, nodal metastasis status was increasing LARS points after CRC surgery in multivariate analysis in our study.

## Data Availability

The raw data supporting the conclusions of this article will be made available by the authors, without undue reservation.
